# Discovery of Novel Genes Encoding Antimicrobial Peptides from the *Pedobacter silvilitoris* Genome with Broad-Spectrum Antimicrobial Activity

**DOI:** 10.3390/ijms26136176

**Published:** 2025-06-26

**Authors:** Woo Young Bang, Jin Hur, Sam Woong Kim

**Affiliations:** 1 Biodiversity Research and Cooperation Division, National Institute of Biological Resources (NIBR), Environmental Research Complex, Incheon 22689, Republic of Korea; 2 Department of Veterinary Public Health, College of Veterinary Medicine, Jeonbuk National University, Iksan-si 54596, Republic of Korea; 3 Agri-Food Bio Convergence Institute, Gyeongsang National University, Jinju 52725, Republic of Korea; 4 FARMIMs Co., Ltd., 55, Dongjin-ro, Jinju 52725, Republic of Korea

**Keywords:** antimicrobial peptide, AMP, cell-free supernatant, genome, transcriptome, *Pedobacter silvilitoris*

## Abstract

The rising prevalence of antibiotic-resistant bacteria demands exploration of alternative antimicrobials. Antimicrobial peptides (AMPs) are a promising group of compounds naturally produced by microorganisms and could serve as potent agents against resistant pathogens. In this study, we evaluated the antimicrobial potential of the cell-free supernatant obtained from *Pedobacter silvilitoris*—a bacterium originally isolated from decomposing wood—and performed comprehensive genomic screening to uncover novel AMP-encoding genes. The supernatant showed strong inhibitory effects against a diverse selection of pathogens. Scanning electron microscopy (SEM) revealed extensive membrane damage, including pore formation in target bacterial cells, suggesting AMP-mediated activity. A genomic analysis identified 11 candidate AMP genes, named PS_AMP1 to PS_AMP11, based on the significant sequence similarity with known AMPs. Transcriptomic profiling further indicated that several candidates are expressed differentially between the logarithmic and stationary growth phases. Functional assays via gene cloning and peptide synthesis confirmed antimicrobial activity against both Gram-stain-negative and Gram-stain-positive bacteria, with PS_AMP11 emerging as the most effective candidate. Our findings demonstrate that AMPs derived from *P. silvilitoris* hold substantial promise as alternative antimicrobial agents. Nonetheless, additional structural optimizations may be necessary to fine-tune specificity and to reduce potential host toxicity before clinical deployment.

## 1. Introduction

The rapid emergence of antibiotic-resistant bacteria poses a significant threat to global public health, necessitating the development of alternative antimicrobial strategies [1,2]. One promising approach is the use of antimicrobial peptides (AMPs)**,** which are naturally produced by various bacteria. Unlike conventional antibiotics, AMPs exhibit a lower rate of resistance evolution [1,3,4], making them a viable alternative for combating multidrug-resistant pathogens, which underscores the potential of AMPs as an effective alternative to conventional antibiotics, particularly in clinical settings where antibiotic resistance is prevalent. Given the urgent need for novel antimicrobial strategies, further exploration of bacterial AMPs is essential. Therefore, this study aims to investigate the genetic basis of AMP production in bacteria, and to evaluate their potential as next-generation antimicrobial agents.

Recent studies have demonstrated that bacterial genomes encode a diverse array of AMPs, including bacteriocins, microcins, and defensins, which play a crucial role in microbial competition and survival. Bacteriocins, produced by both Gram-stain-positive and Gram-stain-negative bacteria, are ribosomally and non-ribosomally synthesized peptides that target closely related bacterial strains [5,6]. Microcins, primarily found in *Enterobacteriaceae*, are small antimicrobial peptides with potent activity against competing bacteria [7,8]. Defensins, although more commonly associated with eukaryotic organisms, have bacterial counterparts that disrupt microbial membranes and inhibit pathogen growth [9,10].

To discover novel AMPs, we recently performed preliminary experiments evaluating the antimicrobial activity of cell-free supernatants obtained from various bacterial strains, and among them, the cell-free supernatant from *P. silvilitoris* was identified to exhibit significant antimicrobial activity against various pathogens. *P. silvilitoris* was first isolated from decaying wood environments in 2015, and was identified to be a bacterial species belonging to the genus *Pedobacter* within the family *Sphingobacteriaceae*, generally known as a group of Gram-stain-negative, rod-shaped, and aerobic bacteria associated with soil, with some species possessing the ability to degrade heparin [11]. However, the research on the physiological and genomic characteristics and ecological role of *P. silvilitoris* remains limited.

In this study, we discovered the genes encoding the AMP candidates through genomic and transcriptomic analyses of *P. silvilitoris* for the first time, and identified several AMPs with significant antimicrobial activity. These results suggest that AMPs found in *P. silvilitoris* have the potential to be utilized as a replacement for conventional antibiotics.

## 2. Results

### 2.1. The Cell-Free Supernatant from P. silvilitoris Exhibits Antimicrobial Activity Against Various Pathogens

**Figure 1:**
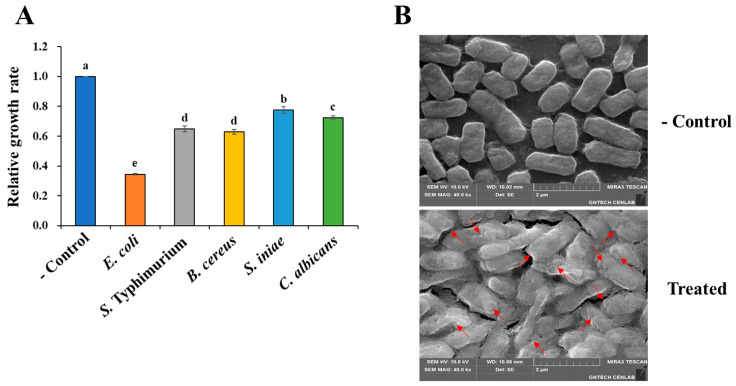
Antimicrobial activity of cell-free supernatant from *P. silvilitoris*. (**A**) Antimicrobial activity of cell-free supernatant from *P. silvilitoris* was examined against *E. coli*, *S.* Typhimurium, *S. iniae*, *B. cereus*, and *C. albicans*, whose relative growth rates were determined. (**B**) For scanning electron microscopy, *E. coli* was treated without (control) or with the cell-free supernatant (treated). The red arrows indicate the pores forming in the *E. coli* membrane. Small letters indicate significant differences among the treated sensitive strains (*p* < 0.05).

We examined the antimicrobial activity of substances produced by *P. silvilitoris*. As shown in Figure 1A, the cell-free supernatant of *P. silvilitoris* showed a broad range of antimicrobial activities against Gram-stain-positive and Gram-stain-negative bacteria, as well as yeast. It specifically inhibited the growth of *Escherichia coli* by about 65%, whereas it inhibited the growth of *Salmonella* Typhimurium, *Streptococcus iniae*, *Bacillus cereus*, and *Candida albicans* by 22% to 35% (Figure 1A). In addition, it was observed by scanning electron microscopy (SEM) that the cell-free supernatant of *P. silvilitoris* disrupts the *E. coli* membranes via pore formation (Figure 1B). In general, typical AMPs have been known to cause cellular lysis through pore formation in the bacterial membrane. Accordingly, these results suggest that the cell-free supernatant of *P. silvilitoris* may include antimicrobial substances, such as AMPs, which mediate the broad range of antimicrobial activities. Therefore, a genome analysis was performed to identify the genes among the hypothetical proteins of *P. silvilitoris* that are AMPs or have high potential for AMP formation.

### 2.2. The P. silvilitoris Genome Includes Various AMP Genes

**Figure 2:**
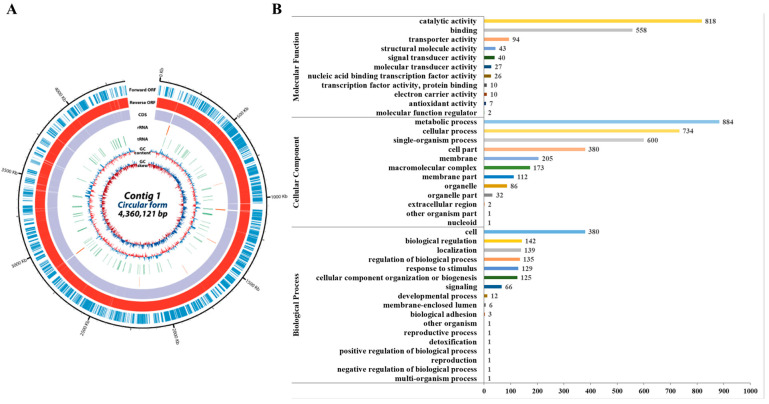
Results of *P. silvilitoris* genomic analysis. (**A**) Overall features of the genome. The outer scale indicates the coordinates in base pairs. The open reading frames (ORFs) are shown on the first two rings; the first ring (blue) is the forward ORF, and the second ring (red) is the reverse ORF. The third and fourth circle shows the ORFs which are colored by gene annotation; the third ring is the forward ORF, and the fourth ring is the reverse ORF. The fifth and sixth circles show rRNA (green) and tRNA genes (orange). In the next circle, the GC content shows whether the GC ratio of the DNA sequence is higher (purple) or lower (deep yellow) than the average. The innermost circle shows GC skew, with light green indicating negative values and deep orange indicating positive values. (**B**) Gene ontology classification of genome. The total genes with Blastall alignment (http://www.ncbi.nlm.nih.gov/books/NBK1762 (accessed on 20 April 2024)) matched against the non-redundant protein database were classified into three main GO categories (biological process, cellular component, molecular function) and 40 subcategories. The vertical-axis shows the detailed names of the subcategories and the horizontal-axis indicates the number of genes in the same category.

The *P. silvilitoris* genome consists of a single chromosome with a total size of 4,360,121 bp and a relatively low GC content of 34.52%. A total of 3792 genes were predicted, of which 2032 were hypothetical proteins (Figure 2A). A Gene Ontology (GO) analysis revealed that the genes associated with molecular functions, cellular components, and biological processes totaled 1635, 3210, and 1144, respectively. The highest count was for the metabolic process (884) under the cellular components, followed by the catalytic activity (818) under the molecular functions (Figure 2B).

**Figure 3:**
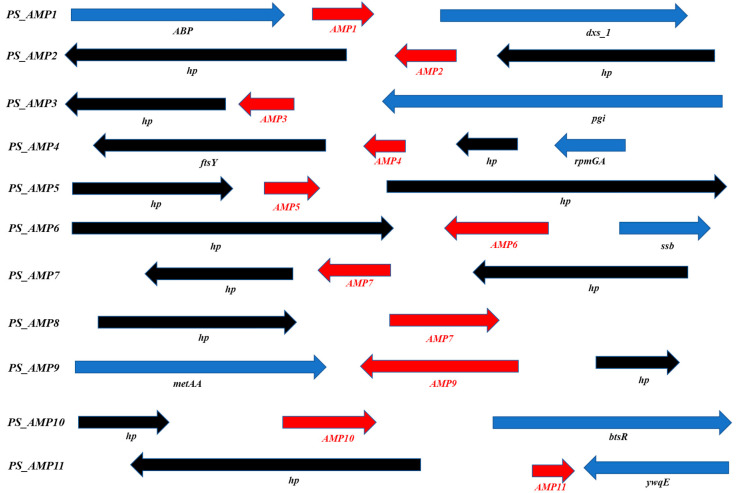
Analyses of predicted *AMP* neighbor genes. *ABP*, putative multidrug export ATP-binding/permease protein; *dxs_1*, 1-deoxy-D-xylulose-5-phosphate synthase; *pgi*, glucose-6-phosphate isomerase; *ftsY*, signal recognition particle receptor FtsY; *hp*, hypothetical protein; *rpmGA*, 50S ribosomal protein L33 1; *ywnH*, putative phosphinothricin acetyltransferase YwnH; *metAA*, homoserine O-acetyltransferase; *btsR*, transcriptional regulatory protein BtsR; *ywqE*, tyrosine-protein phosphatase YwqE.

Moreover, we selected 255 peptides showing a positivity of 50% or more with existing AMPs through an NCBI (https://www.ncbi.nlm.nih.gov/ (accessed on 1 May 2024)) homology analysis on 2032 genes corresponding to hypothetical or uncharacterized proteins. Among these 255 peptides, 16 hypothetical proteins were primarily selected that maintained homology with existing AMPs in various regions and had a relatively small size. From these, a final selection of 11 peptides was made based on showing more than 50% identities/positives with existing AMPs or possessing a signal sequence cleaved by an endopeptidase, and these were designated as PS_AMP1 to PS_AMP11 (Figure 3 and Table 1).

### 2.3. Transcriptomic Results of AMP Candidates from the P. silvilitoris Genome

In order to analyze the expression of the *AMP* candidates selected from the *P. silvilitoris* genome, a transcriptome analysis was performed in the logarithmic growth phase and the stationary phase. As shown in Table 2, the expression of genes 3736 and 3737 in the *P. silvilitoris* culture was observed in the exponential and stationary phases, respectively. Meantime, the 11 *AMP* candidates were identified to be expressed in *P. silvilitoris* (Table 3). Both *PS_AMP*3 and 9 showed higher expression in the logarithmic growth phase than in the stationary phase, which were 3.7 and 3.2 folds higher in the logarithmic growth phase than in the stationary phase, respectively. High expressions of *PS_AMP*5, 7, 10, and 11 were observed in the stationary phase, whereas the remaining *AMP*s, except for *AMP*3 and 9, were highly expressed in the exponential phase, though without statistical significance. In general, it is suggested that genes expressed differently in the logarithmic growth and stationary phases are regulated according to the metabolism and stress response for the adaptation of cells to an environment [12,13]. Therefore, it is suggested that *PS_AMP*3 and 9, exhibiting differential expression between the exponential and stationary phases, play a significant role in the antimicrobial activity of *P. silvilitoris* cell-free culture supernatant.

### 2.4. Antimicrobial Activity of Cloned AMPs from the P. silvilitoris Genome

**Figure 4:**
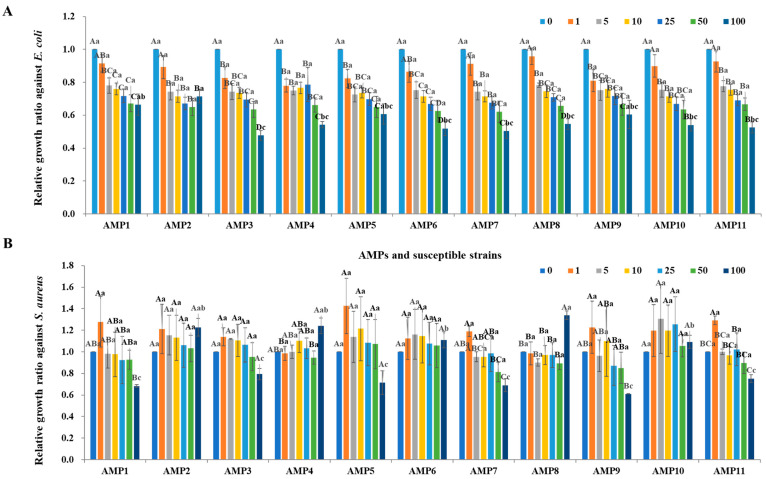
Antimicrobial assays of cloned *PS_AMPs*. The cloned *PS_AMPs* were expressed in *E. coli* DH5α, the cell-free supernatants were separated from the *E. coli* DH5α, and then 1 to 100 μL of cell-free supernatants, as indicated at the top of the graph, were used for the analyses of antimicrobial activity against *E. coli* (**A**) and *S. aureus* (**B**). The *E. coli* and *S. aureus* were adjusted to 10^6^ CFU/mL for the microtiter plate assay. Capital and small letters indicate significant differences among the treated amounts within each AMP and among different AMPs at the same concentration, respectively (*p* < 0.05).

Since the 11 AMP candidates correspond to a part of the sequence of hypothetical proteins, they are expected to be secreted out of the cell through their signal sequences or partially cleaved by peptidase inside the cell in order to exhibit antimicrobial activities. Therefore, to analyze the antimicrobial activity mediated by genes encoding AMP candidates in *P. silvilitoris*, primers were designed to include not only the entire target gene but its upstream and downstream sequences, and these were then amplified by PCR. The PCR-amplified DNA fragments were cloned into the T-easy vector, and the antimicrobial activities were evaluated by the cell-free supernatants of the *E. coli* DH5α strains containing cloned *AMPs* against *E. coli* ATCC 10536, a Gram-stain-negative bacterium, and *S. aureus* ATCC 6538, a Gram-stain-positive bacterium. The cell-free supernatants from the *E. coli* DH5α harboring the *PS_AMP* genes showed significant antimicrobial activity against *E. coli*, whereas they exhibited little antimicrobial activity versus *S. aureus* (Figure 4). These results indicate that the cell-free supernatants from the *E. coli* DH5α harboring the *PS_AMP* genes exhibit antimicrobial activity with higher specificity against Gram-stain-negative bacteria than against Gram-stain-positive bacteria.

### 2.5. Antimicrobial Activity of Synthetic AMPs Originated from the P. silvilitoris Genome

**Figure 5:**
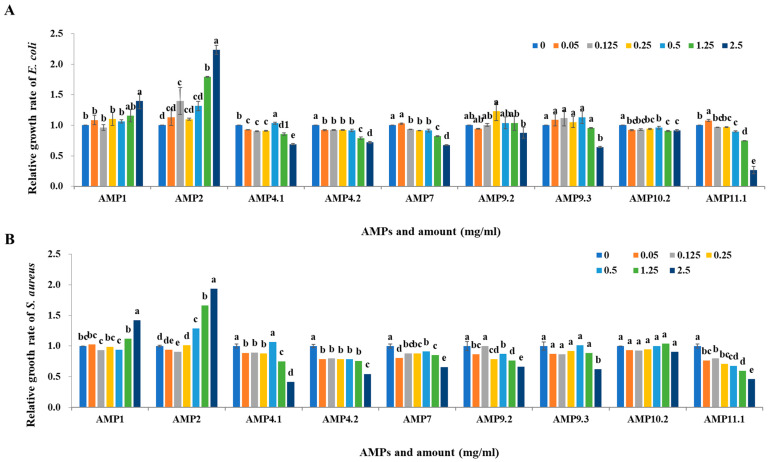
Evaluation of antimicrobial activity of synthetic PS_AMPs. The synthesized PS_AMPs were used for the analyses of antimicrobial activity against *E. coli* (**A**) and *S. aureus* (**B**). The horizontal- and vertical-axes indicate PS_AMP types and the concentration and relative growth rate of *E. coli* and *S. aureus*, respectively. Small letters indicate significant differences among the treated amounts within each synthetic AMP (*p* < 0.05).

To comparatively evaluate the antimicrobial activity of the cell-free culture supernatants from the strains maintaining the cloned *PS-AMP*s and the actually predicted AMPs, only the regions homologous to the preceding AMPs were chemically synthesized (Table 1). Eight of the PS-AMP candidates were synthesized, but PS_AMP3, PS_AMP5, and PS_AMP6 were not synthesized. Noticeably, the hypothetical protein corresponding to PS_AMP4 or PS_AMP9 was identified to possess two or more AMP regions, which were chemically synthesized and named PS_AMP4.1 and PS_AMP4.2 or PS_AMP9.1 and PS_AMP9.2, respectively (Table 1). As a result of evaluating their antimicrobial activity, all synthetic AMPs except for PS_AMP1, 2, and 10 showed antimicrobial activities against *E. coli* (Figure 5A) and, in particular, PS_AMP11 showed the highest antimicrobial activity with an MIC50 of 1.9 mg/mL. The synthetic AMPs also showed antimicrobial activity against *S. aureus* (Figure 5B) and, similarly, PS_AMP4.1 and AMP11 showed higher antimicrobial activities with an MIC50s of 2.2 and 2.0 mg/mL, respectively. Taken together, our genomic and transcriptomic analyses revealed that *P. silvilitoris* produces at least 11 hypothetical proteins, containing regions that function as AMPs, which was further supported by the antimicrobial activities of the cloned or synthesized PS_AMPs.

### 2.6. Structural Models of PS_AMPs

**Figure 6:**
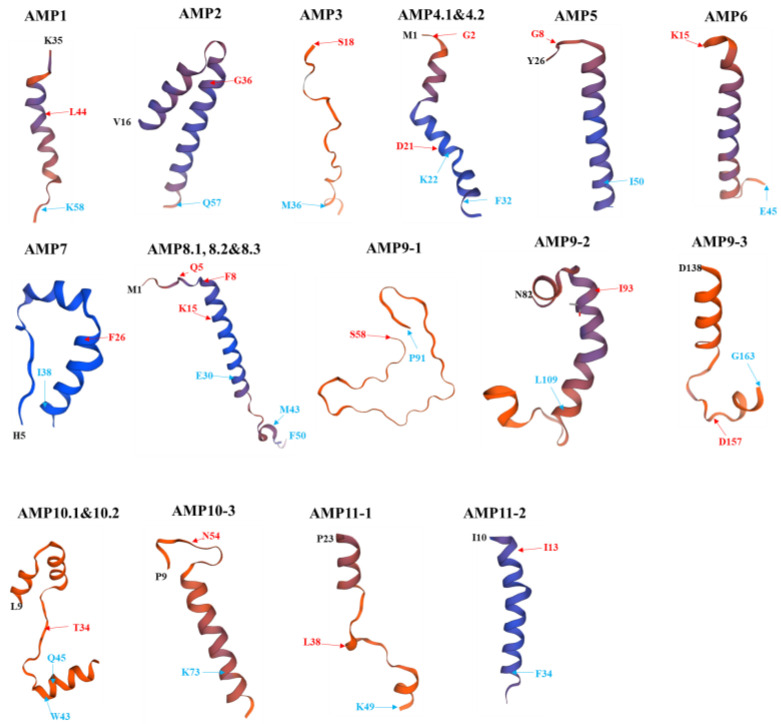
Structural models of PS_AMPs. Most of the PS_AMPs form helix structures. Black letters indicate the start site of a query sequence, red arrows and letters represent the start site of an AMP part, and blue arrows and letters indicate the finish site of an AMP part or a query sequence. Their structures were predicted by the SWISS-MODEL server (https://swissmodel.expasy.org/interactive (accessed on 4 April 2025)).

The three-dimensional structures of the 11 AMPs described in Table 1 were modeled. As shown in Figure 6, AMP1, 2, 4.1, 4.2, 5, 6, 7, 8.1, 8.2, 8.3, 9.2, 10.3, and 11.2 were found to form alpha-helical structures. By contrast, AMP3 and AMP9.1 appeared to adopt a random coil conformation, while AMP9.3, 10.1, 10.2, and 11.1 exhibited a mixed structure of random coil and alpha-helix. Since AMP3, which is predicted to contain a signal peptide, adopts a random coil structure, its antimicrobial activity is expected to differ from the typical behavior of conventional AMPs.

Among the synthesized peptides, AMP4.1, which showed high antimicrobial activity, is a pleurain-K1 analog and forms an alpha-helix structure in the region corresponding to D21–F32. AMP4.2, with an estimated MIC_50_ of 3.0 mg/mL, was slightly less active compared to AMP4.1 (2.22 mg/mL), and corresponds to the G2–K22 region of the aurein-1.2 analog (Table 1 and Figure 6). The structure of AMP4.2 exhibits a slightly modified alpha-helix, which is presumed to account for its slightly lower activity compared to AMP4.1. AMP11.1 was evaluated as the most effective peptide among the synthesized ones against both Gram-stain-positive and Gram-stain-negative bacteria. In the structural model of AMP11.1, five amino acids at the C-terminal region were automatically omitted during prediction. While the E43–K49 region maintained an alpha-helix, the L38–K42 region exhibited random coil conformation. It is presumed that the alpha-helical region plays a direct role in membrane pore formation.

## 3. Discussion

Conventional antibiotics usually target specific cellular processes (e.g., cell wall or protein synthesis), which can lead to the rapid selection of resistant mutants [14,15,16]. By contrast, AMPs generally exert their effects by disrupting bacterial membranes—a broader target that is less prone to resistance development [4]. Many AMPs achieve this through the formation of helical structures that facilitate pore formation [17,18], a mechanism that is supported by our SEM observations (Figure 1B) and structural models of the PS_AMPs.

The cell-free supernatant of *P. silvilitoris* exhibited significant antimicrobial activities against general pathogenic bacteria, such as *E. coli* and *S. aureus*. The antimicrobial activity of the cell-free supernatant, based on scanning electron microscopy observations, is presumed to be primarily induced through the formation of holes in the cell membrane, and to proceed by gradually causing cell disintegration, implying strongly that the antimicrobial activities may be mediated by peptide substances like AMP. Interestingly, the three-dimensional structure models of PS-AMPs from *P. silvilitoris* showed that most of them form alpha-helix structures to maintain net positive charges. It is suggested that the PS_AMPs, to form net positive charges and helix structures, may effectively cause cellular lysis by pore formation in the cell membrane with the alpha-helix via attachment to the negatively charged bacterial cell envelope, as do typical AMPs [17,18]. In summary, the results revealed that the AMPs from the *P. silvilitoris* genome exhibited bactericidal activity and supported the reason why the biogenics of *P. silvilitoris* showed antimicrobial activity.

Noticeably, the ability of the engineered *E. coli* strains expressing these *PS_AMP* genes to inhibit bacterial growth further reinforce the role of these peptides in antimicrobial defense. For example, the presence of predicted signal peptides in the AMP candidates suggests a conventional secretory pathway, while the absence in others implies alternative release mechanisms (e.g., through cell lysis). For example, The EcDBS1R6 shows antimicrobial activity after its signal peptide is removed by an endopeptidase [19], whereas the E protein of bacteriophage phiX174 induces lysis from within the cell [20]. Therefore, we suggest that the PS_AMP3, predicted to carry a signal sequence, may be delivered outside the cell via its signal sequence, while other PS_AMPs not predicted to maintain a signal sequence, may be released either by lysis from inside the cell or from cells that have undergone natural lysis. However, since AMP3 exhibits a random coil structure, it is presumed that its antimicrobial activity may occur via the carpet mechanism, which differs from the typical pore-forming mode of action observed in conventional AMPs [2].

Among the synthesized AMPs, PS_AMP11.1 showed the best antimicrobial activity, and exhibited 53% identities and 76% positive with the E17KG peptide, known to have antimicrobial activity against both Gram-stain-positive and negative pathogens. In addition, The PS_AMP11 has a hydrophobic ratio of 41%, which is similar with the typical cationic AMPs, but a net charge of +4, which is slightly higher than the typical AMPs. Although the structure of AMP11.1 uniquely exhibited a combination of alpha-helix and random coil, and it had a high net positive charge, it showed notably higher antimicrobial activity compared to other AMPs with predicted alpha-helical structures. Further studies are considered necessary to investigate this aspect.

While AMPs are generally considered less likely to induce resistance, prolonged exposure to these peptides may eventually lead to adaptive changes in bacterial membranes (e.g., alterations in surface charge, cell wall thickening, or enhanced efflux) [4,21]. Furthermore, potential challenges, such as nonspecific toxicity to host cells and difficulties in large-scale production, warrant careful optimization [22,23]. Approaches such as fine-tuning the net charge, combining AMPs with traditional antibiotics, or modifying the balance of hydrophilic and hydrophobic residues might improve both efficacy and safety [24,25]. For industrial production, recombinant expression systems in microorganisms, like *E. coli* or yeast, could offer viable scaling strategies.

Currently, clinically investigated AMPs, such as defensins, LL-37, and polymyxins, serve as benchmarks. Defensins, which maintain a broad range of antimicrobial effects against bacteria, viruses, and fungi, have been applied to increase their stability and to mitigate resistance [26]. LL-37, which studied as a treatment for skin infections and wound healing, has been developed with modifications and combination therapies to enhance its antimicrobial effect [27]. Polymyxins, used to treat infections, such as *Pseudomonas aeruginosa* and *Acinetobacter baumannii*, have been studied to mitigate their toxicity and to increase efficacy [28]. Although some of our candidates demonstrated promising activity, further engineering and optimization of the PS_AMP peptides will be essential to fully realize their clinical potential.

The findings from this study suggest that certain genes with homology to known AMPs may be expressed and processed to exhibit antimicrobial activity. While this study raises the possibility of antimicrobial activity in these genes, it does not clearly explain the underlying reasons. Further research is needed to understand how AMP candidates with sequence homology within genes can develop antimicrobial properties. For instance, studies should investigate whether peptides expressed through artificial cloning retain similarity to the homologous regions identified, as well as how they behave after expression. Additionally, further investigation is warranted to determine how peptides, like AMP11.1, which maintain a partial alpha-helix structure, can exhibit such high levels of activity.

In summary, this study identified 11 previously uncharacterized AMP candidates from the genome analysis of *P. silvilitoris*. Antimicrobial activity was evaluated through both cloning, ensuring inclusion of their native promoters and terminators, and peptide synthesis. Among them, AMP11.1 exhibited the most potent antimicrobial activity. AMP11.1 forms a partial alpha-helix structure and has a net positive charge of +4. Further research is needed on potential modifications and mechanisms of action to enhance the activity of AMP11, including AMP11.1, for improved industrial applicability.

## 4. Materials and Methods

### 4.1. Medium and Bacterial Strains Employed for This Study

The media used in this study were R2A, TSB, LB, and YM (Sigma-Aldrich, St. Louis, MO, USA). If required, the solid medium was made by supplementation with 1.5% agar. A *P. silvilitoris* strain with the culture number, NIBRBAC000002732, was obtained from the National Institute of Biological Resources (Incheon, Republic of Korea) and was cultured for 24 h at 30 °C in an R2A medium. *Salmonella* Typhimurium (KCTC12401), *Escherichia coli* (ATCC 10536), *Bacillus cereus* (ATCC 11778), *Streptococcus iniae* (KCTC3657), *Staphylococcus aureus* (ATCC 6538), and *Candida albicans* (KCTC7122) were purchased from KCTC (Korean Collection for Type of Cultures, Daejeon, Republic of Korea). The *C. albicans* was incubated at 25 °C and the above bacteria, except for the *C. albicans*, were incubated at 37 °C.

### 4.2. P. silvilitoris Genome Sequencing

Genomes were isolated from *P. silvilitoris* using a genome preparation kit (Wizard Genomic DNA purification kit, Promega, Madison, WI, USA), and 5 μg of purified genome was used for library preparation. A SMRTbell library was constructed according to the manufacturer’s protocol for the SMRTbell™ Template Prep Kit 1.0 (PN 100-259-100, Pacific Biosciences, Menlo Park, CA, USA). Small DNA fragments of less than 20 kb of SMRTbell template were removed using a BluePippin Size Selection system (Sage Science, Beverly, MA, USA) for the large-insert library. The constructed library was subjected to quality control using the Agilent 2100 Bioanalyzer (Agilent Technologies, Santa Clara, CA, USA). Sequence analysis oligomers were annealed to the SMRTbell template, and then DNA polymerization was carried out using the DNA/Polymerase Binding kit P6. This polymerase–SMRTbell-adaptor complex was spotted into a SMRT cell. The SMRTbell library was sequenced with 1 SMRT cell using C4 chemistry (DNA sequencing Reagent 4.0) with the MagBead OneCellPerWell v1 Protocol (Insert Sizes 20 kb, movie time 1 × 240 min). Images were recorded in the SMRT cell for 240 min using the Pacific Biosciences (PacBio RS II) sequencing platform. This resulted in 116,823 and 1,148,362,395 base pairs of long reads following subread filtering.

De novo assembly was performed using a hierarchical genome assembly process (HGAP, Version 2.3) workflow containing consensus policing with Quiver (https://github.com/lukeping/GenomicConsensus (accessed on 3 April 2024)). As the estimated genome size was 4,382,491 bp and the average coverage was 103×, we performed error correction based on the longest of about 30× seed bases (150,011,291 bp), with the rest being shorter reads, and then assembled this with error-corrected reads. As a result of the HGAP process, 4,382,491 bp N50 contig and 4,382,491 bp total contig lengths were obtained by the polishing process. Finally, since bacterial genomes and plasmids are typically circular forms, each contig was examined using MUMmer 3.5 (https://mummer4.github.io/ (accessed on 16 April 2024)), and one of the self-similar ends was trimmed for manual genome closure.

Putative gene coding sequences (CDSs) from assembly contigs were identified using Glimmer v3.02 (https://ccb.jhu.edu/software/glimmer/index.shtml (accessed on 18 April 2024)), and open reading frames (ORFs) were obtained. These ORFs were explored using Blastall alignment (http://www.ncbi.nlm.nih.gov/books/NBK1762 (accessed on 20 April 2024)) in a non-redundant protein database (nr). GO annotation was assigned to each ORF using Blast2GO software (Version 5.2) through a maximum hit analysis of BLAST results. Additionally, ribosomal RNAs and transfer RNAs were predicted using RNAmmer 1.2 (https://services.healthtech.dtu.dk/services/RNAmmer-1.2/ (accessed on 23 April 2024)) and tRNAScan-SE 1.4 (https://lowelab.ucsc.edu/tRNAscan-SE/ (accessed on 25 April 2024)).

### 4.3. Bioinformatic Analysis for Identification of AMPs from the P. silvilitoris Genome

Peptides with a similarity (identities/positives) of more than 50% to existing AMPs or possessing a signal sequence cleaved by an endopeptidase were selected as AMP candidates using CAMP (http://www.camp.bicnirrh.res.in/campHelp.php (accessed on 1 May 2024)) or SignalP 5.0 (https://services.healthtech.dtu.dk/services/SignalP-5.0/ (accessed on 2 May 2024)), respectively. Their amino acid and nucleotide sequences were subsequently used for peptide synthesis and gene cloning, respectively.

### 4.4. Exploration of AMPs Gene Expression Through Transcriptomic Analysis

Total RNA extraction was performed using the AccuPrep^®^ Bacterial RNA Extraction Kit (Bioneer, Daejeon, Republic of Korea). RNA purity was measured using 1 μL of total RNA in a NanoDrop1000 spectrometer (Thermo Fisher Scientific, Waltham, MA, USA). The measured RNA integrity number (RIN) value was checked using the Agilent 2100 Bioanalyzer (Agilent Technologies) to confirm the quality of the total RNA. A Nugen Universal Prokaryotic RNA-seq kit (Part Number 0363-32, NuGEN Technologies, San Carlos, CA, USA) was used for the fabrication of total RNA sequencing libraries, which were carried out according to the manufacturer’s protocol. Total RNA (300 ng) was used to synthesize first and second strand cDNA with selective primers. Fragmentation of 200 bp size was carried out by sonication using a Covaris S220 (Covaris, Woburn, MA, USA) in a microtube. In Strand Selection II, the rRNA was removed according to the bacterial specifications using the AnyDeplete technique with the AnyDeplete® probe (Tecan, Männedorf, Switzerland). The libraries were amplified using PCR, and the amplified amount was confirmed using capacitive electrophoresis (Bioanalyzer, Agilent Technologies). qPCR was performed with SYBR Green PCR Master Mix (Applied Biosystems, Waltham, MA, USA), and RNA sequencing was performed using an Illumina Novaseq 6000 system (Illumina, San Diego, CA, USA).

### 4.5. P. silvilitoris AMPs Gene Cloning and Peptide Synthesis

General gene cloning was performed using the method of Sambrook et al. (1989) [29]. Candidate *AMPs* obtained through the analysis of the genome and transcriptome were designed and synthesized with primers for PCR amplification (Table 4; Genotech, Daejon, Republic of Korea). After amplifying each *AMP* gene using this primer set and a PCR PreMix (AcuPower PCR PreMix, Bioneer), gel extraction was performed. Eluted DNA fragments were ligated to the T-easy vector (Promega) and transformed into *E. coli* DH5α (Invitrogen, Carlsbad, CA, USA) using the heat-shock method using calcium chloride. Cloned *AMPs* were identified using restriction enzyme cleavage and nucleotide sequencing (Applied Biosystems). To analyze the antimicrobial activity of the cloned samples, *E. coli* transformed with the T-easy vector alone was used as the control group.

Amino acid sequences in regions with homology to predicted AMPs were obtained, and these sequences were synthesized as artificial peptides (Table 1; Cosmogenetech, Seoul, Republic of Korea).

### 4.6. Evaluation of Antimicrobial Activity of Cell-Free Supernatants and Synthetic Peptides

The cell-free supernatant of *P. silvilitoris* or all *E. coli* DH5α strains containing cloned *AMPs* was collected after 24 h of culture and filtered through a 0.2-μm syringe (mixed cellulose esters (MCE), Merck, Darmstadt, Germany), and finally the cell-free supernatant was used for the evaluation of antimicrobial activity. The cell-free supernatant from the *E. coli* DH5α, harboring only the plasmid vector without the *PS_AMP* genes, was confirmed as a negative control to have very little effect on antimicrobial activities. The synthesized AMPs were suspended at a concentration of 10 mg/mL in distilled water and then used for antimicrobial activity.

The antimicrobial activities against *S.* Typhimurium (KCTC12401), *E. coli* (ATCC 10536), *B. cereus* (ATCC 11778), *S. iniae* (KCTC3657), *S. aureus* (ATCC 6538) and *C. albicans* (KCTC7122) were analyzed using the microtiter plate method and expressed as minimal inhibition concentration (MIC); the bacteria pre-cultured overnight (O/N) were mixed with 10^6^ CFU/mL with the cell-free supernatant or the synthetic peptides, the reaction mixtures were added to the microtiter plate, and finally the reactivity was observed by the O/N culture at 37 °C. The obtained A600 values were normalized by arbitrarily setting the control group, which did not contain cell-free supernatant, to 1.0. The relative activity of the treated samples was then expressed as a ratio of their A600 values to that of the control.

Relative growth rate = A/B A: the treated sample A600 value B: non-treated (control) A600 value

### 4.7. Scanning Electron Microscopy (SEM)

The *E. coli* cells treated with the cell-free supernatant of *P. silvilitoris* were fixed with a volume fraction of 2.5% glutaraldehyde (Sigma-Aldrich, Milwaukee, WI, USA) for 24 h at 4 °C. The samples were rinsed with a sterile PBS buffer twice, and then dehydrated with 30%, 50%, 70%, 80%, 90%, and 100% (*v/v*) graded ethanol, successively, with 15 min incubation at each concentration. The samples were dried at room temperature and sprayed with gold coating before the SEM observation.

### 4.8. Statistical Analysis

The collected data were analyzed using the PROC ANOVA procedure of the SAS program (ver. 9.2; SAS Institute Inc., Cary, NC, USA). Mean values that differed at the level of 5% significance were verified using Duncan’s multiple range test (DMRT).

## Figures and Tables

**Table 1:** PS_AMPs of *P. silvilitoris* applied for this study.

AMPs Name	ORF Sequences *	Homologous AMPs		Synthesized AMPs			
		Homolog Name	Identities/Positives (%)	Amino Acid Seq.	Amino Acid No.	pI	MW
PS_AMP1	MSQFVLMGLFFFPLVVSVLTIKDVFENQGLDNGKKLMWIATII**LLPLVGAIIYFFFGK**SKQL	LAMP_Experimental_3178	47/73	LLPLVGAIIYFFFGK	15	8.59	1698.12
PS_AMP2	MEDKFDYQNYNWLYYVIGLVCGLLVGYAVEGHIIT**GAILGLLTGGLFQFLINKSREQ**EA	Melittin	46/67	GAILGLLTGGLFQFLINKSREQ	22	8.75	2375.80
PS_AMP3	***MMKKPIISLFVGGLLSLSLLNACDSNKNA*EPEVTQM**ETLQDSLEQQETALNKDIENLQKSMQDLDQEFKKNN	Granulosusin-E1	40/66	MKKPIISLFVGGLLSLSLLNACDSNKNAEPEVTQM	35	5.93	3762.45
PS_AMP4	M**GLFDFFKKKDKTTDEQEALDKGLEKTKDNFF**PK	Pleurain-K1	58/83	DKGLEKTKDNFF (PS_AMP4.1)	12	6.12	1441.60
		Aurein-1.2	43/57	GLFDFFKKKDKTTDEQEALDK (PS_AMP4.2)	21	4.93	2503.79
PS_AMP5	MSQRKPYNHNTSYGRRKIREQSEKYYE**GLPPEEKSSHNLLGILIMVIICI**IMFLILGADGFLKWGTR	HsAp3	39/65	GLPPEEKSSHNLLGILIMVIICI	23	5.4	2490.06
PS_AMP6	MTFQDR**KDKGKDWDKVKEKREELHERDEKIANEPKAVLKQDQETEQE**KEE	AVP1829	27/71	KDKGKDWDKVKEKREELHERDEKIANEPKAVLKQDQETEQE	41	5.09	4978.46
PS_AMP7	MNIYHFFPLPHPSPRNIIWQAKNRW**FVKEVLPQLKEII**SVHI	Kasstasin	62/77	FVKEVLPQLKEII	13	6.14	1555.92
PS_AMP8	MARS**QETFSKKENEKKRLKKRQEKQQKKEERRESSTGGGLENMMAYVDEF**GNITDTPPDPIRKKKEIDASTIEIGIPKQEAEDLTATKKGKIEFFNDSKGFGFIKEDNTQEKFFVHVNGLTEEVREGDKVSFELEKGLKGMNAVNVKRI	Magainin-2	28/64	KKRLKKRQEKQQKKEERRESSTGGGLENMMAYVDEF (PS_AMP8.1)	36	9.77	4343.95
		AVP1828	26/56	QETFSKKENEKKRLKKRQEKQQKKEERRESSTGGGLENM (PS_AMP8.2)	39	9.9	4709.28
		Alpha-benincasin	30/83	FSKKENEKKRLKKRQEKQQKKEE (PS_AMP8.3)	23	10.09	2975.44
PS_AMP9	MLQNLINLVKENAGESIINNPSVPNEKNDKAIEVTGNSIIETLKNAVAGGDI**SQLKSLFNQEPEAAKTSTLANNAQNNVVANLIAKIGLSP**E**IATKIASTVVPLVMSKL**VSKTNDPNNSSFNIQDILGNLAGGNGGFDVGSILSGINSANDKNKKG**DVLGKLGGLFGK**	Opistoporin-1	44/54	SQLKSLFNQEPEAAKTSTLANNAQNNVVANLIAKIGLSP (PS_AMP9.1)	39	8.22	4095.62
		D83	59/76	IATKIASTVVPLVMSKL (PS_AMP9.2)	13	10	1771.23
		Ponericin-G7	75/83	DVLGKLGGLFGK (PS_AMP9.3)	12	8.59	1203.45
PS_AMP10	MFFVTYNSLNKAIRYFFVDPYIEQYEKENGNFA**TWWKTQGTPWQQ**FINPIAFS**NAIKGANMVVWIAVTIKLFK**LWYQKRKAALYAELNFLKSQVHPHFLFNTLNNLYALTLNQSPKAPQVVMGLSEILRYMLYECDKETISLKKN	Pep-1-K	67/67	TWWKTQGTPWQQ (PS_AMP10.1)	12	8.41	1546.70
		PK-12	70/70	TWWKTQGTPW (PS_AMP10.2)	10	8.41	1290.44
		Brevinin-2-RA17 peptide precursor	55/70	NAIKGANMVVWIAVTIKLFK (PS_AMP10.3)	20	10.3	2216.76
PS_AMP11	MEFILPGIGIPEI**ITILIALIIPIAIITMIVLYFRKNLKLKKEQIELLKKIANK**	E17KGG	53/76	LKLKKEQIELLKKIANK (PS_AMP11.1)	17	10	2037.56
		CAMPST453	38/58	ITILIALIIPIAIITMIVLYFRKNLK (PS_AMP11.2)	26	10.29	2997.89

* The bold sequences represent putative AMP sequences, and the italic sequences with underlines in the PS_AMP3 a indicate the signal sequences. The putative AMP and signal sequences were predicted using CAMP (http://www.camp.bicnirrh.res.in/campHelp.php (accessed on 1 May 2024)) and SignalP 5.0 (https://services.healthtech.dtu.dk/services/SignalP-5.0/ (accessed on 2 May 2024)), respectively.

**Table 2:** Expression profiling of transcriptomes from *P. silvilitoris*.

Sample	Total Reads	Mapped Reads	Mapping Rate %	Count (>0)	Exp. (>1)
Pedobacter_1	51,645,840	16,626,807	32.19	3736	3736
Pedobacter_2	57,465,126	17,422,082	30.32	3737	3737

Pedobacter_1; exponential phase, Pedobacter_2; stationary phase; Reference; *Pedobacter* 3737 genes from *P. silvilitoris.*

**Table 3:** Expressions of candidate *AMPs* in *P. silvilitoris*.

AMPs Name	Pedobacter_1c	Pedobacter_2c	Pedobacter_1e	Pedobacter_2e	Fold Change (PS1/PS2)	*p* Values
AMP1	3698.7	5874.8	344.072	218.014	1.578211	0.7023
AMP2	5050.79	5853.11	469.85	217.209	2.163124	0.1024
AMP3	4591.43	3140.14	427.118	116.531	3.665274	0.0015
AMP4	4352.97	9476.85	404.935	351.686	1.151411	0.18465
AMP5	1007.97	3463.72	93.766	128.539	0.729475	0.93455
AMP6	4059.62	3269.95	377.646	121.348	3.112091	0.50425
AMP7	2923.71	9205.92	271.978	341.632	0.796114	0.18795
AMP8	18734.59	38260.64	1742.786	1419.853	1.227441	0.8038
AMP9	1869.67	1455.25	173.926	54.004	3.220613	0.016
AMP10	400.45	1727.74	37.252	64.117	0.581	0.42205
AMP11	808.69	4104.77	75.228	152.328	0.493855	0.87705

Pedobacter_1: exponential phase; Pedobacter_2: stationary phase; c: read count; e: normalized.

**Table 4:** Oligonucleotides of *P. silvilitoris* applied for this study.

Forward Primers		Reverse Primers	
Name	Nucleotide Seq. (5′ → 3′)	Name	Nucleotide Seq. (5′ → 3′)
PS_AMP1-F	ATTGCCCGTTCCCTGTTAAG	PS_AMP1-R	TTGCACCAAAATGACCTCCG
PS_AMP2-F	CCAAGGCAATGATCTGTCAC	PS_AMP2-R	GAGCGAGCCTGTTAATTTAGG
PS_AMP3-F	TCCACCTATCGCTTACCTTC	PS_AMP3-R	ATCGCTCCAATCCTTAACGC
PS_AMP4-F	TAGCGCTTGCACCTCTTCTC	PS_AMP4-R	TCACCTAAAACAGGTGCTTACT
PS_AMP5-F	TGGTTATATGGCTCGGGAAC	PS_AMP5-R	TGGATTGTCAGCATTGTCGG
PS_AMP6-F	AACAGTGCTACTCGTTTCGC	PS_AMP6-R	GTAAGTGCTACTCCGCTACA
PS_AMP7-F	TTAACCTGCTGTGTGCTAGG	PS_AMP7-R	TTTGCTAGCCGCCTAAGGTA
PS_AMP8-F	GCTGGAGTAGCTTGGTTAATG	PS_AMP8-R	TTATGAAGAGAGCGTCTGTACA
PS_AMP9-F	ATAAGGGACTGCCTATTGCC	PS_AMP9-R	CATATTCTGACTGCATACCACC
PS_AMP10-F	AGTTACCTTACTTGGGTTAACC	PS_AMP10-R	CGATACTGTTGGCAACGGTA
PS_AMP11-F	GAGCGTTTTAACACGCCATAC	AMP11-R	TACGGCTGCTACTTTCAGTTAA

## Data Availability

The original contributions presented in this study are included in the article. Further inquiries can be directed to the corresponding author.

## Abbreviations

The following abbreviations are used in this manuscript:

AMP	Antimicrobial peptide
SEM	Scanning electron microscopy
